# Neoadjuvant Chemotherapy prior to Radical Prostatectomy for Patients with High-Risk Prostate Cancer: A Systematic Review

**DOI:** 10.1155/2013/386809

**Published:** 2013-02-21

**Authors:** Stavros Sfoungaristos, Vasileios Kourmpetis, Eleftherios Fokaefs, Petros Perimenis

**Affiliations:** Department of Urology, Patras University Hospital, 26500 Patras, Greece

## Abstract

High-risk prostate cancer represents a pretentious clinical problem since a significant number of its patients will relapse and progress after radical prostatectomy. Neoadjuvant chemotherapy may be valuable since its efficacy in hormone-resistant prostate cancer has been established. In this paper, we report studies of neoadjuvant chemotherapies that have been used in high-risk patients prior to radical prostatectomy. Even though the results regarding the prognostic surrogates are not significant, the effects on clinical and pathological outcomes are promising, while toxicity in most of the studies is in the expected field.

## 1. Introduction

Prostate cancer (PCa) represents the most common malignancy in men and the second leading cause of death in Western countries [1]. According to the Surveillance, Epidemiology and End Results (SEER) program, for year 2012, 241740 new cases of PCa will be diagnosed in the United States of America, while 28170 men will die from the disease [1]. Implication of PCa screening in combination with recent advantages in diagnostic strategies has led to an increased detection of patients of early age with low-stage PCa. However, a treatment failure reaching 35%–40% will occur after primary therapy, and a kind of adjuvant treatment will be required [2, 3]. In an effort to identify those patients with high risk for recurrence and progression after treatment and with adverse pathological features after primary surgical management, in terms of extracapsular disease or seminal vesicles and lymph node invasion, stratification models have been introduced to categorize patients into risk groups [4, 5]. Risk stratification is based upon PSA, histological grade (Gleason score), and clinical stage. High-risk patients, meaning those with PSA levels >10 ng/mL, Gleason score >7, or clinical stage ≥T2c, have a significant higher possibility of biochemical relapse after treatment [4, 6]. Even in the era of PSA screening, 20%–35% of patients with newly diagnosed PCa are classified as high risk for adverse prognosis [4, 7].

The optimal treatment for these patients has not been standardized. Radical prostatectomy (RP) remains an option for these patients [28]. However, an increased incidence of treatment failure has been observed, and many of them will progress after RP mainly due to distant micrometastasis. For this reason, a multimodal therapeutic approach has been proposed for this certain group of patients in order to destroy remnant of cancerous cells at the operation bed or occult distant metastases.

In the present work, we concentrate on the use of neoadjuvant chemotherapeutic agents to RP. We reviewed PubMed/Medline database to identify studies which have evaluated neoadjuvant chemotherapeutical protocols in patients with high-risk localized or locally advanced PCa. Data were extracted from eligible studies and assessed for quality, inclusion criteria, number of participants, and outcomes.

## 2. Neoadjuvant Chemotherapy

Several randomized studies have evaluated the clinical, pathological, and prognostic outcomes and safety of neoadjuvant chemotherapy prior to radical prostatectomy (Table 1).

### 2.1. Docetaxel

Docetaxel belongs to taxane group of chemotherapeutic drugs with proved efficacy in castration-resistant PCa [29]. Docetaxel demonstrates a significant improvement in survival and has been recognized as the standard therapy after failure of hormone therapy. Based on this standard knowledge, many investigators have used docetaxel, either alone and or in combination with hormone therapy and/or other chemotherapeutics, in neoadjuvant fashion prior to RP in patients with high-risk PCa to evaluate its efficacy in this group of patients.

#### 2.1.1. Docetaxel Monotherapy

Dreicer et al. [8] performed a phase II trial of docetaxel monotherapy administered on a weekly basis for 6 weeks before RP. The study included 29 patients with PCa of clinical stage ≥T2b, PSA ≥15 ng/mL, or Gleason score ≥8 and no evidence of metastatic disease. A statistically significant reduction in the prechemotherapy versus postchemotherapy mean of PSA level was observed (*P* < 0.03) with 79% of patients to experience some reduction and 24% to experience a more than 50% reduction in PSA level. No unexpected toxicities and no intraoperative complications were reported. After a median followup of 23 months, 20 patients were cancer-free.

In a later publication of the same study cohort with main objective to determine the histological and molecular changes after neoadjuvant treatment, the authors reported that docetaxel may be a significant promoter of apoptosis due to an increased expression of p53 and Bcl-2 that was detected after immunohistochemical staining of surgical specimens [9]. Furthermore, they reported that after a median follow up of 49.5 months, 12 patients (43%) remained clinically and biochemically free of disease with no additional therapy required, while the rest of the patients had experienced biochemical progression.

The clinical, pathological and molecular effects of neoadjuvant docetaxel were the purpose of study conducted by Febbo et al. [10]. They enrolled 19 patients with high-risk localized PCa to receive weekly docetaxel for 6 months prior to RP. Toxicity was mild to moderate. Although no pathological complete responses were observed, there was a significant decline of >50% concerning PSA measurement in 11 of 19 patients and tumor volume reduction >25% in 17 patients as it was shown in endorectal MRI. In the molecular level, microarray analysis showed that altered androgen metabolism may be partially involved with the noted declines in PSA and may be a mechanism for chemotherapy resistance.

#### 2.1.2. Docetaxel Plus Hormone Therapy

Docetaxel combined with complete androgen blockade has been evaluated for efficacy and toxicity.

Chi et al. [11] in a phase II multicenter study enrolled a total of 72 patients with high-risk localized prostate cancer to receive complete androgen deprivation therapy combined with docetaxel. Eight men did not complete therapy because of toxicity and other reasons. Although pathological downstaging was observed, no significant (3%) pathological complete response rates were noticed. Positive surgical margins were identified in 27% of patients. After a median follow up of 42.7 months, a recurrence-free survival rate of 70% (44 patients) was observed.

Based on the same concept, Mellado et al. [12] enrolled a total of 57 patients with PSA >20 ng/mL and Gleason score ≥7 (4 = 3) to receive a neoadjuvant combination of docetaxel and complete androgen deprivation. The pathological complete response rate was 6%, failing to reach the primary endpoint of the study which was 10% pathological complete responses. However, this rate was closed to the expected one after hormonal therapy alone. The regimen was well tolerated with no significant grade 3 or 4 toxicities to be noticed.

#### 2.1.3. Docetaxel Plus Estramustine

This combination represents the most studied regimen in neoadjuvant protocols.

In 2011, a phase II trial conducted by Kim et al. [13] studied patients with high-risk localized or locally advanced PCa. The study cohort was divided to receive neoadjuvant docetaxel and estramustine chemotherapy either prior to treatment with RP (12 patients) or prior to external beam radiation therapy (10 patients). A PSA reduction of >25% was achieved by 21 patients. However, no pathological complete responses were noticed, while the 2-year progression-free survival was 45% after a median followup of 24 months.

The same combination regimen was evaluated for tolerance and feasibility by an earlier trial of Hussain et al. [14]. The investigators included patients with ≥T2b PCa, PSA ≥15 ng/mL, and/or Gleason score >7 to undergo either RP (10 patients) or radiation therapy (11 patients). Negative surgical margins were found in 7 patients and positive postchemotherapy and preradiotherapy biopsies in 2 patients of the radiation arm. Pathological complete responses were not observed. The treatment was well tolerated with neutropenia to be found in 9 patients and 2 cases to develop deep vein thrombosis.

There are 3 publications in the literature which have studied preoperative combination of docetaxel, estramustine, and hormone therapy. In the first one, conducted by Prayer-Galetti et al. [15], docetaxel and estramustine were combined with triptorelin. After a median follow up of 53 months, 8 patients out of 21 (42%) were disease-free, while responding patients had an 85% disease-free survival rate at 5 years. Three patients had a complete response, and 16 of them had partial response.

Sella et al. [16] studied 22 patients with PCa at high risk for local and systemic relapse after RP. They received docetaxel and estramustine plus complete androgen blockade (goserelin plus bicalutamide). Disease-free survival was noted in 54.5% of patients after a median follow up of 23.6 months.

Similarly, Narita et al. [17] used docetaxel and estramustine plus leuprorelin and bicalutamide. A pathological complete response was observed in 2 of 18 patients enrolled, with 77.8% being disease-free after a median follow-up of 18 months.

#### 2.1.4. Docetaxel Plus Other Agents

Recently, Garzotto et al. [18] evaluated the pathological and survival outcomes in 54 high-risk patients after neoadjuvant combination chemotherapy (docetaxel + mitoxantrone) for 16 weeks followed by RP. Negative surgical margins were attained in 67% of cases. After a median follow-up of 63 months, the authors reported that the combination regimen is feasible with 47.4% of patients to be disease-free with no clinical or biochemical evidence of progression. The recurrence-free survival at 2 years and 5 years was 65.5% and 49.8%, respectively. In addition to this, clinical and molecular predictors (pretreatment serum PSA, lymph node involvement, and postchemotherapy tissue vascular endothelial growth factor expression) for early recurrence were identified.

In a phase II trial, Womble et al. [19] studied docetaxel plus ketoconazole as neoadjuvant treatment of 22 patients with high-risk, clinically localized prostate cancer. Main endpoints were biochemical recurrence rate and tolerance of therapy. The results showed acceptable toxicity with 16 patients completing all 4 cycles of therapy. However, there was no significant improvement in biochemical recurrence rate over standard (30%–40% free of recurrence) after definite local therapy. At a median follow up of 18 months, only 36% of patients who underwent RP remained free of recurrence.

Friedman et al. [20] combined docetaxel with capecitabine. The regimen was administered to a total of 15 patients with high-risk PCa followed by local therapy, 11 of them underwent RP. The combination regimen was well tolerated, but neither pathological complete response was observed nor significant PSA responses.

Ross et al. [21] were the first to report results from neoadjuvant docetaxel and an antiangiogenic substance, specifically bevacizumab. The authors performed a multicenter phase II trial, and they enrolled 41 patients with PSA >20 ng/mL or PSA velocity >2 ng/mL/y, clinical stage >T3, and Gleason score >7. Patients were treated with docetaxel 70 mg/m^2^ every 3 weeks for 6 cycles and bevacizumab 15 mg/m^2^ every 3 weeks for 5 cycles. The primary endpoint was partial response by endorectal MRI. Although there were no complete pathological responses, 29% of patients achieved a >50% reduction in tumor volume, and 22% achieved a >50% posttreatment decline in PSA.

### 2.2. Paclitaxel

Paclitaxel chemotherapy drug is classified within the taxane group and is used for the treatment of hormone refractory PCa. Paclitaxel works by stiffening the microtubules that make up the inner skeleton of a cell. Once the microtubules are locked into place, the cells, which require malleability to divide successfully, crumble and die during cell division.

Since paclitaxel is an efficient therapeutic option for patients with castration-resistant PCa, 2 recent studies deal with this regimen either alone or in combination with other drugs as a neoadjuvant treatment prior to RP.

Shepard et al. [22] evaluated the efficacy of nab-paclitaxel, a novel nanopeptide-based formulation. They enrolled a total of 19 patients who completed 2 cycles of 150 mg/m^2^ nab-paclitaxel weekly for 3 weeks followed by RP. The regimen was well tolerated. There were no significant perioperative complications. Ten patients had grade 3 and 1 patient had grade 4 neutropenia with no febrile neutropenia. Although a significant preoperative decrease in serum PSA was noticed after chemotherapy (*P* < 0.001), there were no pathological complete responses.

In a phase I/II study, Konety et al. [23] enrolled 36 high-risk patients who received a combination neoadjuvant therapy of paclitaxel, carboplatin, and estramustine in addition to complete androgen deprivation. The authors reported a rate of positive surgical margins close to 22%, while the biochemical recurrence-free survival was 45% after 29 months of median follow up. Deep vein thrombosis was the most frequent complication reaching 22%.

### 2.3. Other Chemotherapeutics

The feasibility of estramustine in combination with etoposide as neoadjuvant therapy in patients with locally advanced high-risk PCa was examined and confirmed in a phase II study conducted by Clark et al. [24]. Out of 18 studied patients, grade 3 toxicities were observed in 5 patients, and 1 patient experienced a grade 4 pulmonary embolism. Although the pathological analysis demonstrated a higher rate than expected of organ-confined disease, an undetected serum PSA level prior to prostatectomy was achieved only in 50% of patients. At a median follow up of 14 months, all patients with negative lymph nodes were disease-free.

In another study by Garcia et al. [25], the biological effects and safety of granulocyte-macrophage colony-stimulating factor and thalidomide administered preoperatively in patients with localized PCa were examined. No pathological complete responses were observed, and regarding the molecular changes, no significant post-treatment tumor T-cell and dendritic cell infiltration was noted.

In a 2000 study by Pettaway et al. [26], 33 patients with high-risk PCa were recruited to receive ketokonazole, doxorubicin alternating with vinblastine, estramustine, and androgen ablation followed by RP. The goal of achieving a 20% rate of pT0 status was not achieved, but 20 of the 29 patients who completed therapy remained with no disease recurrence after a median follow up of 13 months.

Exisulind is a cyclic guanosine monophosphate phosphodiesterase inhibitor, and its analogs have been shown to induce apoptosis in vitro in many cancer cell lines including PCa cell lines without affecting normal human cells. Weight et al. [27] divided the studied patients to the treatment group (*n* = 44) to receive 375–400 mg oral exisulind daily for 4 weeks and then to undergo RP and the control group (*n* = 49) to undergo RP per routine. The drug was well tolerated, and there were no serious adverse events. However, there were no significant effects of exisulind on biomarkers of cell death (bcl-2, Bax, caspase 3, and PTEN) between biopsy specimen and post-treatment RP specimen, which was the primary endpoint.

Vuky et al. [30] used docetaxel plus gefitinib in 31 patients with PCa. RP specimen pathology demonstrated residual carcinoma in all cases. Twenty-nine (94%) patients achieved a clinical partial response, including 35% of patients who demonstrated radiographic improvement on MRI. Grade 3 toxicities were noticed in 19% of patients. One patient experienced grade 4 toxicity.

The value of neoadjuvant imatinib, a platelet-derived growth factor receptor inhibitor, in men with high-risk localized PCa was studied by Mathew et al. [31]. The authors reported no complete pathological responses but a 53% progression-free survival after 39 months of follow-up in 36 patients who received neoadjuvant leuprolide plus docetaxel plus imatinib.

Ayala et al. [32] assessed the safety of administering bortezomib, a proteasome inhibitor, in 40 patients with locally advanced, high-grade PCa who were undergoing RP, and they evaluated the pathological changes in RP specimen. One patient had grade 3 gastrointestinal toxicity. An antitumor activity was manifested expressed by increased tumor cytopathic effect, drops in serum PSA in some patients, and an increase in tumor apoptosis.

## 3. Discussion

Although radical surgery represents the primary therapeutic option for patients with clinically localized disease, the indications have been expanded to promote RP as an acceptable option for patients with high-risk local disease and even locally advanced disease [28, 33]. PCa exhibits a wide spectrum of clinical behaviors, ranging from small, nonsignificant tumors with a minimal possibility of progression after radical therapy or even surveillance to aggressive types with increased potential to metastasize and cause disease-related death. Consequently, there is a considerable variation in the risk of biochemical and clinical progression among patients. In an effort to define those PCa patients with high risk for progression, several prognostic nomograms have been proposed to assess the potential risk. Even in the PSA era and the timely diagnosis of prostate cancer, 20%–35% of patients with newly diagnosed cancer are still classified as high risk for adverse prognosis, defined as preoperative PSA >20 ng/mL, biopsy Gleason score >8, and/or clinical stage >T2c [4, 34]. Prognostic findings following RP for disease recurrence and disease-specific survival include extracapsular disease, positive surgical margins, pGleason score ≥8, seminal vesicle, and lymph node invasion. Although there is no consensus regarding the optimal treatment of men with high-risk PCa, RP remains an option for this category of patients [28]. However, this certain group of patients harbor the danger of clinical metastases and death from PCa. Early biochemical relapse following RP may occur in these patients which negatively impacts the prognosis. Thus, a multimodal treatment fashion appears as a reasonable and attractive way to counsel such patients.

Although a high-risk population with clinically localized PCa is likely to optimally benefit from neoadjuvant treatment, the published trials have used heterogeneous criteria to characterize patients as high risk making difficult the cross-comparison of the studies. Thus, the formation of a universally accepted risk stratification is of great research and ethical importance which will enable future studies to evaluate adjuvant or neoadjuvant therapies in a specifically and clearly characterized high-risk category of patients. Such an evolution will optimize the exported results, will assist the comparison between different therapeutic protocols, and will enable the extraction of reliable and important conclusions.

Previous studies have established the efficacy of chemotherapy for patients with hormone refractory PCa [29, 35–38]. Based on this, many investigators have evaluated the value of neoadjuvant chemotherapy prior to RP. The goal of such concept is that neoadjuvant treatment may shrink advanced disease and/or lymph node metastases. Thus, resection of the complete tumor may be feasible, leaving behind at the operation bed no cancerous material. Furthermore, tumor downstaging may have a prognostic significance. In addition, neoadjuvant chemotherapeutic protocols may offer assessment of chemosensitivity in vivo of several potential molecular determinants and the evaluation of response in the RP surgical specimens. This may allow rapid evaluation of promising new agents.

In this paper, we have presented a number of studies that used chemotherapy neoadjuvantly to RP. Summarizing the results, there are some important conclusions. Neoadjuvant chemotherapy promotes a beneficial effect on the pathological findings. Most of the studies report a decrease in the positive surgical margins and extraprostatic disease rates, and some of them reveal a benefit in the biochemical-free and overall survivals. Of course, it is of great importance to notice that all of the studies used survival surrogates, meaning biochemical-free and systemic progression-free survivals, to estimate the prognostic outcomes. In general, it seems that the application of neoadjuvant therapy in patients undergoing RP does not provide a significant advantage in disease-free and overall survivals over prostatectomy alone. On the other hand, it may substantially improve the local pathological adverse control.

Similar to the neoadjuvant chemotherapy, hormone therapy has been widely studied as a neoadjuvant treatment prior to RP. Most of the studies have exported positive results on the pathological outcomes, in terms of positive surgical margins and/or extracapsular extension; however, no benefit has been reported in survival outcomes (biochemical-free survival and overall survival). This is the main difference between neoadjuvant chemotherapy and hormone therapy. The first one has proved benefits in survival surrogates, while the later did not reach any benefit.

The main concern about the usage of neoadjuvant chemotherapy remains the adverse events. In general, most of the published works report a well-tolerated and safe profile of the different chemotherapeutic agents, with only estramustine-based regimens to produce notable risks of deep vein thrombosis and pulmonary embolism.

There is ongoing interest in the value of neoadjuvant chemotherapy in patients with PCa. Although current knowledge is based mainly on phase II trials, the results so far are promising, especially regarding the effects on the postoperative pathological findings, and most of the studies report a decrease in positive surgical margins and extraprostatic disease rates in patients who received neoadjuvant chemotherapy. These findings merit further exploration through randomized control studies.

Regarding the given cytotoxicity of these agents, the majority of them have been generally described as safe and well tolerated with only estramustine-based regimens producing notable risks of deep vein thrombosis and pulmonary embolism.

## 4. Conclusions

Neoadjuvant chemotherapy prior to RP for patients with high-risk PCa may influence the clinical and pathological outcomes in a positive manner. Its value for prognosis remains to be examined through randomized control trials.

## Figures and Tables

**Table 1:** Trials of neoadjuvant chemotherapy to radical prostatectomy.

Authors, references	No. of patients	Regimens	Toxicity	Followup	Results
Dreicer et al., [8]	29	Docetaxel	NUT	23 m	20 patients disease-free
Magi-Galluzzi et al., [9]	28	Docetaxel	NUT	49.5 m	43% biochemical-free
Febbo et al., [10]	19	Docetaxel	NUT (fatigue and taste disturbances)	n/a	>50% of ↙PSA in 58% of patients, no pathological complete response
Chi et al., [11]	64	Docetaxel plus CAB	4 withdrawals	42.7 m	2 pathological complete responses, 70% recurrence-free survival
Mellado et al., [12]	57	Docetaxel plus CAB	10.1% of patients did not complete the therapy	35 m	6% pathological complete responses
Kim et al., [13]	12 RP/22 RT	Docetaxel plus estramustine	1 withdrawal	24 m	>25% of ↙PSA in 21/22 of patients, 45% progression-free survival
Hussain et al., [14]	10 RP/11 RT	Docetaxel plus estramustine	2 patients with grade 3 deep venous thrombosis, 1 patient with grade 4 neutropenia	n/a	Positive surgical margins in 3 patients
Prayer-Galetti et al., [15]	21	Docetaxel plus estramustine plus LHRH analogue	NUT	53 m	26% positive surgical margins, 58% organ-confined disease, 42% disease-free, 15% complete response, 80% partial response
Sella et al., [16]	22	Docetaxel plus estramustine plus CAB	NUT	23.6 m	54.5% disease-free survival
Narita et al., [17]	18	Docetaxel plus estramustine + CAB	No grade 3 or 4 toxicities	18 m	11% pathological complete response, 77.8% disease-free survival
Garzotto et al., [18]	22	Docetaxel plus mitoxantrone	Grade 4 leukopenia, neutropenia, hyperglycemia	63 m	Recurrence-free survival after 2 y and 5 y was 65.5% and 49.8%
Womble et al., [19]	22	Docetaxel plus ketoconazole	4 withdrawals, 16 patients with grade 3/4 toxicities	18 m	8/18 biochemical-free
Friedman et al., [20]	15	Docetaxel plus capecitabine	NUT	n/a	No pathological complete responses
Ross et al., [21]	41	Docetaxel plus bevacizumab	3 withdrawals, 3 patients with grade 3 neutropenia	n/a	>50% of ↙PSA in 22% of patients, no pathological complete responses
Shepard et al., [22]	18	Paclitaxel	10 patients with grade 3 and 1 patient with grade 4 neutropenia	n/a	95% PSA decrease, no pathological complete responses
Konety et al., [23]	36	Paclitaxel plus carboplatin plus estramustine plus CAB	Deep vein thrombosis in 22% of patients	29	45% biochemical recurrence-free survival
Clark et al., [24]	18	Estramustine plus etoposide	1 patient with grade 4 pulmonary embolism, 2 patients with grade 3 deep venous thrombosis	14	↗Rate of organ-confined disease, thromboembolism in 3 patients
Garcia et al., [25]	28	Thalidomide plus GM-CSF	NUT	32	5/28 patients had recurrence
Pettaway et al., [26]	33	Doxorubicin plus ketoconazole plus estramustine plus vinblastine plus CAB	NUT	13	17% positive surgical margins, 20/29 patients with no disease recurrence
Weight et al., [27]	93 (control study)	Exisulind	NUT	n/a	No effects on biomarkers of cell death

GS: Gleason score; CAB: complete androgen blockade; RP: radical prostatectomy; RT: radiation therapy; NUT: no unexpected toxicities; n/a: not available; GM-CSF: granulocyte-macrophage colony-stimulating factor; m: months; y: years; LHRH: luteinizing hormone-releasing hormone.
